# Communication from H. A. J. New Orleans

**Published:** 1855-05

**Authors:** 


### EDITORIAL.

NEW ORLEANS, March 19th, 1855.

DR. DAVIS:

Dear Sir,—As I am spending a few days in this city, I have determined to avail myself of the opportunity to visit the hospitals and become acquainted with its medical advantages. A few mornings since I had the pleasure of following Drs. Stone and Nott through their wards in the Charity Hospital. In the wards of Dr. Nott were a large number of consumptives, the most of whom had contracted the disease in a Northern climate, and had come here to wear out the remainder of life. So far as I could see, no treatment was pursued. One case which came in just before our visit, was hastily examined; the ear placed beneath the scapula for a moment, and with two rude thumps the patient was passed, the Dr. remarking that "there were cavities, and that it was a hard case." Dr. Nott had the kindness to show me a very fine specimen of diseased bone, the enlarged shaft of a femur enclosing a sequestrum, while the head and neck of the bone were very much atrophied, although healthy in appearance. The disease had been of long standing, and I learned from the young man who made the post mortem, that the ilium was diseased.

Dr. Stone showed me a case in which he had recently removed a malignant tumor of the lower lip. Two incisions were made parallel to each other, and extending from the angles of the mouth to the hyoid bore. The flap was dissected loose, and slid up, the diseased parts having been removed, and retained in position by twisted sutures. The operation was successful, their being at present but little deformity. I also saw in the surgical wards a case of hydrocele, under treatment. The method adopted was by opening freely with a lancet or scalpel, the sac, evacuating the fluid contents, and keeping the orifice patent by the introduction of a tent of lint. Dr. Stone tells me that this is his usual couse, as he has found it more successful than injections, and the effects more under the control of the surgeon. We doubt it.

Dr. Stone has also recently tied the gluted artery for traumatic aneurism. It is reported at length, with remarks, in the New Orleans Medical News and Hospital Gazette. A boy, ten years of age, on the 28th of December last received a penetrating wound from a narrow bladed knife in the gluteal region. The wound healed kindly, and the patient was out in a few days. On the evening of the third of January, sharp lancinating pains were felt in the gluteal region, extending during the next day to the knee and ancle; on the fifth, his physician, Dr. Randall, of Galveston, Texas, discovered a large pulsating tumor in the wounded region, which, in spite of compression and every means used to prevent it, continued to increase. Dr. R. proposed to ligate the gluteal artery, but his colleagues not consenting, the patient was brought to this city and placed under the care of Dr. Stone, who operated by laying open the tumor, removing the clotted blood, and placing a ligature around the artery at the bottom of the wound.

The operation was perfectly successful, the patient being discharged on the 17th day. Ligature of the gluteal artery is certainly a rare operation. Its depth beneath the surface, and in traumatic aneurism the difficulty of finding it in the infiltrated tissues, would certainly be sufficient to deter most surgeons from making the attempt, except as a last resort.

Dr. Stone's patients, even those with vascular excitement, were mostly on tonics and stimulants, a course of treatment to which observation and experience sooner or later conducts most practitioners in the South-west, in spite of the theories of inflammation and its treatment by antiphlogistics. In several cases of fracture the dressings were very simple.

I have just attended the commencement of the medical department of the University of Lousiana. The address to the class by Dr. Jones consisted in an enumeration of the names of great men who have been connected with the profession of medicine. The effect on our own mind was to make us feel that we were in good company, and that, however much quackery may have done to disgrace us, these nobles of our race have done infinitely more to adorn and dignify our noble science and our philanthropic art.

The Dr. intimated that he had a work in preparation in which these magnates of the profession would be catalogued.

The facilities for medical study are as good here, I am quite satisfied, as they can be in New York or Philadelphia. From ten to fifteen thousand patients are prescribed for annually in the Charity Hospital alone. In addition, the Marine Hospital is accessible to students, thus furnishing the most ample opportunities for the study of clinical medicine and surgery, and of pathological anatomy.

I spent a very pleasant evening with the members of the New Orleans Academy of Sciences—an institution which has been growing up here for the last two or three years. The academy is in a flourishing condition, and is doing much towards stimulating the young men of the city to scientific investigations. It must be evident to every one acquainted with the natural resources and prospective development of the Mississippi Valley, that the American character, with its institutions, its science and its literature, must grow up here in the West. Literary and scientific organizations, however small may have been their beginning, are, therefore, of vast importance in the effects which they may have in giving direction to future efforts. We should have in our own city something of this kind. The natural history of Northern Illinois, its geology, minerology, and meteorology are but little known. This, in addition to the questions of general interest, would furnish abundant material to interest our young men at least, and would open the way for more extended scientific inquiries in the future.

In looking over a late number of the *Compte Rendus*, in the possession of Professor Riddell, I find the following, which, from its physiological interest, I send you for publication. For the translation I am indebted to my friend, Prof. W. P. Riddell, of the chemical laboratory of the University of Louisiana.

---

*On the Manner in which Small Corpuscles pass from the Intestines to the Interior of the Chyliferous and Sanguineous Vessels.* By Messrs. F. MARFELS and J. MOLESCHOTT, of Heidelberg.

We have proved, by a great number of experiments, made on the frog, that small corpuscles, with a smooth surface, (the molecules of the black matter of the choroid of the eye and the sanguineous corpuscles of the ewe and of the ox,) pass from the cavity of the intestines into the sanguineous capillaries, of the mesentery and of the heart. Repeated experiments and observations, made on the frog, on the ox, on the rabbit, on the dog, and on man, have demonstrated to us that the particles pass from the stomach and the intestine into the cells which cover the mucus of these organs, and which, as Mr. Brucke, the illustrious physiologist, of Vienna, has justly remarked, are only enclosed by a soft mucus, which is permeable to small molecules of fat. After having passed out of this cellular system, the corpuscles enter the depressions of the villous coats of the mucus, and then into the freely opened roots of the chyliferous vessels which Mr. Brucke has described, and which only take their origin beyond the villosities in the mucus of the intestines. We have since found the molecules of the black matter of the eye, which we had mixed with the meat and milk which formed the nourishment of our dogs, in the chyliferous vessels of the mesentery, and in the thoracic canals of these animals. The channel which these corpuscles follow in traversing from the intestinal cavity into the sanguineous vessels, has been established in all its extent by direct observation.

The passage of the black matter of the eye into the epithelial cells of the intestine also occurs after death, especially if it be favored by a temperature of about 86° Fah., and a pressure of from nine to ten centimres of mercury.

This permeability of the epithelial cells of the digestive organs is not a general property of cells; it is wanting in the corpuscles of the blood of the frog, as well as in the polygonal cells of the human tongue.

We would also add that the digestion of the greater portion of fat, as has been well observed by Mr. Brucke, should be regarded as operating in virtue of a mechanical transport, and not as a simple solution—the digestive juices only enjoying, in fact, the power of suponifying but a small proportion of the fat which we digest.

---

To Dr. Choppin, one of the editors of the New Orleans Medical News and Hospital Gazette, and your friend Dr. Fenner, as well as to Professors Riddell and Stone, I am under many obligations for their kindness in rendering my stay in the city both pleasant and profitable. H. A. J.

